# An Asset-Based Examination of Contextual Factors Influencing Nutrition Security: The Case of Rural Northern New England

**DOI:** 10.3390/nu17020295

**Published:** 2025-01-15

**Authors:** Claire H. Ryan, Caitlin Morgan, Jonathan G. Malacarne, Emily H. Belarmino

**Affiliations:** 1 Food Systems Program, University of Vermont, Burlington, VT 05405, USA; claire.ryan@uvm.edu; 2 Food Systems Research Unit, USDA Agricultural Research Service, Burlington, VT 05405, USA; caitlin.morgan@usda.gov; 3 School of Economics and Maine Agricultural and Forest Experiment Station, University of Maine, Orono, ME 04469, USA; jonathan.malacarne@maine.edu; 4 Department of Nutrition and Food Sciences, University of Vermont, Burlington, VT 05405, USA; 5 Gund Institute for Environment, University of Vermont, Burlington, VT 05405, USA

**Keywords:** nutrition security, rural, assets, qualitative research

## Abstract

Background/Objectives: Rural communities face a disproportionate burden in terms of diet-related health challenges and have been identified as a target for the U.S. Department of Agriculture’s nutrition security initiatives. In this paper, we adopt an asset-based approach and use the Community Capitals Framework to examine the characteristics that support nutrition security in rural communities, using rural northern New England as a case study. Methods: We conducted focus groups and interviews with 32 food and nutrition professionals in Maine, New Hampshire, and Vermont in 2023 and 2024 to explore the contextual factors that influence nutrition security in rural communities. We coded the data for community assets and mapped the identified assets into the seven dimensions of the Community Capitals Framework: built capital, cultural capital, financial capital, human capital, natural capital, political capital, and social capital. Results: The participants described assets in all dimensions of the Community Capitals Framework except built capital. The specific assets discussed were related to local food production (natural and cultural capital), coordination between food system stakeholders and strong social networks (human and social capital), regional political commitments to food security and nutrition (political capital), and the strong seasonal tourist economy present in some communities (financial capital). Conclusions: Rural communities remain under-studied in the literature regarding nutrition, and little is known about how to advance healthful eating in rural contexts. An asset-based approach was helpful for identifying existing resources that enhance rural nutrition security and may provide an opportunity to characterize and disseminate strategies to advance rural health equity.

## 1. Introduction

In 2022, the U.S. Department of Agriculture (USDA) expanded on its previous food security and nutrition goals to focus on nutrition security, which is defined as “consistent and equitable access to healthy, safe, affordable foods essential to optimal health and well-being” [1]. An integral part of this initiative is advancing equity, which recognizes that there are numerous structural factors that may prevent Americans from accessing and consuming a healthy diet, including structural racism [2], poverty, inequality, discrimination, and stigma [3]. Rural communities are recognized by this initiative as one group that may have a disproportionately difficult time consuming a healthy diet, but the factors that can support nutrition security for rural residents are not specified [3,4].

The focus on rural communities is relevant given rural health disparities. Heart disease [5], diabetes [5,6], and hypertension [7] are all more prevalent among rural populations compared to their urban counterparts, and higher rates of premature death have been identified in rural areas [8]. Differences in nutrition security in rural areas may be a contributing factor to these health disparities, given the importance of diet to the etiology of numerous chronic conditions [9].

National evidence shows a higher prevalence of food insecurity in rural areas (15.4%) when compared to suburban areas (11.7%), but not metropolitan areas (15.9%) [10]. Although rural diet quality remains under-studied [11], national and regional analyses have found that rural diets deviate substantially from dietary recommendations [12,13]. While additional research is needed to compare the diets of those living in rural and nonrural areas [14,15], food environments are known to vary substantially by geography [16,17,18] and are a determinant of eating behaviors [19]. For example, the retail food environment of rural areas differs significantly from that of urban areas, with potentially strong effects on residents’ abilities to access health-promoting foods. Larger grocery stores, which have been found to provide greater variety [20], higher quality [21], and lower-priced foods [22], are more likely to be found in nonrural areas [23]. Additionally, the average distance to retail food stores is greater in rural compared to nonrural areas [17,24]. The key themes relevant to rural food environments and diets were recently summarized by Seguin et al. [11].

Northern New England is among the most rural regions of the country, with 61% of Vermont and Maine residents and 40% of New Hampshire residents living in areas classified as rural [25]. The food security rates in each state are above the national averages [10]. Further, statewide data suggest that residents of all three states have higher rates of meeting daily intake recommendations for vegetables than the national average, and residents of New Hampshire and Vermont have higher rates of meeting daily intake recommendations for fruits than the national average [26]. The region’s high rate of rural residents and comparatively favorable food security and dietary intake data highlight northern New England (NNE) as an area with strengths relevant to rural nutrition security, despite challenges.

Employing an asset-based approach [27] to assessments of rural areas serves to counteract the deficit-based approaches that are typically used when discussing rural health [28]. Asset-based approaches are grounded in the strengths of individuals and institutions in a community and their social ties with each other, thereby reinvigorating local governments, businesses, and other organizations to build institutional power to affect positive change in a community [29]. Few studies have examined the strengths of rural communities regarding healthy food access [30,31].

One framework for identifying and organizing rural community assets is the Community Capitals Framework (CCF) [32]. This framework organizes a community’s assets into seven interrelated types of capital: natural, cultural, human, social, political, financial, and built. To better understand how an asset-based approach could potentially be used to support nutrition security initiatives in the United States, the present study applies the CCF to the food system in rural NNE. Through the identification of specific assets that support nutrition security in the region, we will fill a gap in the evidence of rural community assets for nutrition and begin to elucidate a model of factors that may enhance diets in rural communities.

## 2. Materials and Methods

Six focus groups (*n* = 22) and ten interviews were conducted between October 2023 and April 2024 with nutrition and food system experts from Maine, New Hampshire, and Vermont. Participants were recruited using purposive and snowball sampling. Organizations involved in nutrition, food, or health across the three states were requested to share the recruitment email with their staff and other contacts. Individuals who expressed interest in participation were asked to complete a brief survey asking about their demographic information and recommendations for other potential participants. Interviews were pursued with individuals with state-level expertise on rural food systems and diets. Focus groups included 2 to 5 participants and lasted 40–85 min. Interviews lasted 25–60 min. Participants received a $25 gift card. All research study protocols and materials were approved by the University of Vermont Institutional Review Board (#STUDY00002454).

The focus group discussions explored how rural communities’ culture, economic status, and local organizations influence diet quality; the circumstances that influence residents’ access to healthy food; and how participants would categorize rural communities in their state based on their local economy and population status [33]. After the first focus group, the discussion guide was refined to improve the discussion flow. An interview guide was created to uncover how environmental, cultural, social, and economic factors influence nutrition security in the region; the perceived relative importance of economic versus noneconomic factors in shaping regional nutrition security; and how participants would categorize rural communities in their state [33]. Discussions began with a brief introduction from the interviewer, outlining the purpose of the research and introducing the focus on assets rather than deficits. See both discussion guides in the Supplementary Materials (S1 and S2).

The first author conducted all the focus groups and interviews over Microsoft Teams, and recordings were transcribed verbatim using the application’s automatic transcription feature. Transcripts were checked for accuracy by comparing them to the audio recordings.

NVivo (version 14 for Mac) was used to examine and code the data. A template analysis approach was used for coding [34]. An a priori codebook was formulated and organized into topical areas representing contextual domains that might relate to nutrition security such as those outlined by the Food and Agriculture Organization [35,36], references to marginalized communities, and those regarding rurality [33]. Within each topical area, codes were suggested to represent related discussion topics. The codebook was applied to three of the transcripts by the first author and a research assistant. They then compared codes to come to an agreement on the utility of the a priori themes and identify additional emergent themes. The codebook was updated based on its applicability to these transcripts, including removing and consolidating some codes and adding others. All the initially coded transcripts were recoded using the updated codebook. Double coding was used for a subset of the data. Once consistency was established between the coders, the first author coded the remainder of the data and coding was reviewed by the research assistant. Following coding, the data were analyzed by re-reviewing subthemes within each code against the CCF and mapping the identified assets onto the framework [32]. For example, text coded under “Land access” was examined for specific relevance to natural capital; however, intersections with other types of capital were also mapped. A list of codes is presented in the Supplementary Materials (S3). All subthemes, including assets, were captured and tracked in code memos, along with illustrative quotes.

## 3. Results

Table 1 presents the personal and professional characteristics of the food system experts who participated in the research. Most identified as women (93.75%) and were non-Hispanic White (91.63%). Ages ranged from 24–66 with an average age of 45. The participants worked in a variety of food- and nutrition-related roles, including roles in public health/nutrition programs (34.38%), food or farm non-profit organizations (28.13%), University Extension programs (18.75%), food hubs (9.38%), as clinical dietitians (6.25%), and one as an Indigenous community leader (3.13%).

### 3.1. Rural Assets Identified by Food System Experts

Participants were accustomed to applying a deficit-oriented lens when thinking about factors that shape local diets. Two participants explained the advantages of examining rural residents’ nutrition security using an asset focus.

“I just think that there can be areas that are… ‘economically poor’ by these capitalistic defined standards that might be very, very rich in other ways that aren’t defined, that aren’t measured, that aren’t given room in the conversation, aren’t given room in like policymaking conversations.”

Food hub staff member, Vermont.

“I hope if anything gets taken away from my interview and I hope others are saying the same thing, too, is that… nutritious food is so much more than the macro-, micro-… nutrients and calories that are in the food and whether it’s in the grocery store or not. And rural diets consist of so much more that is often left untracked that I would imagine lead to a way more nutritious diet than is given credit for.”

Food systems network staff member, Vermont.

**Figure 1:**
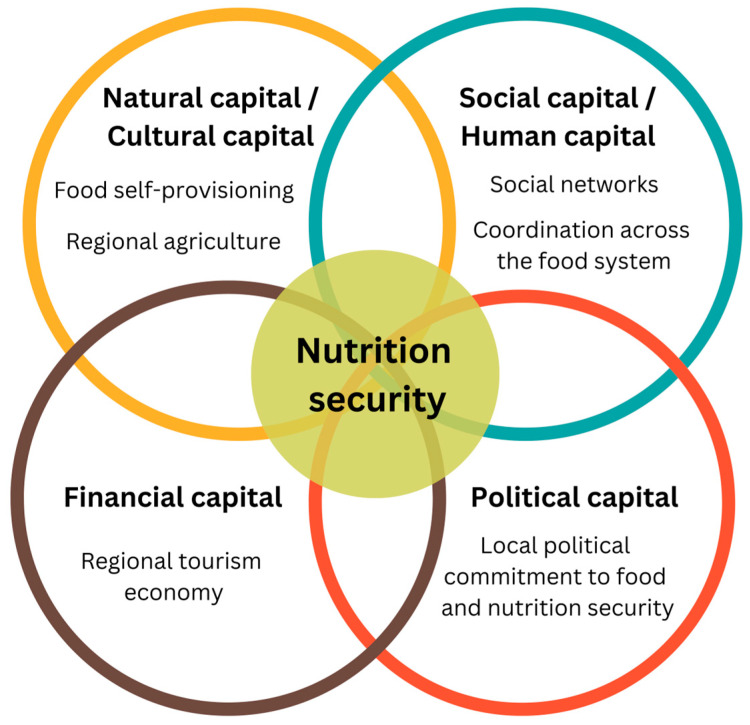
Assets supporting rural nutrition security in northern New England, mapped onto the Community Capitals Framework.

Through the focus groups and interviews, the food system experts identified diverse assets that positively contribute to nutrition security in the rural communities in which they work. Below, we use the CCF to present these assets (Figure 1), which align with each dimension of the framework except built capital. Reflecting the inherent relationships between types of capital, the figure displays overlap between the dimensions of the CCF. Notably, participants rarely highlighted specific individuals or communities as “positive deviants” with respect to rural nutrition security. Rather, they described existing resources in rural NNE that are and can be leveraged for nutrition.

### 3.2. Natural Capital/Cultural Capital

Despite the short growing season in Northern New England (approximately May–October [37]), the participants described both food self-provisioning (FSP) and regional agriculture as important to nutrition security. Food self-provisioning was described as a way in which residents can build their resiliency by producing their own foods and was noted as a significant cultural activity. Participants discussed seven types of FSP: hunting, fishing, gardening, canning, foraging, aquaculture, and raising backyard chickens. For some rural marginalized communities, such as New American and Indigenous groups, engaging in FSP can support access to culturally relevant foods that are not available through traditional markets.

“We have a lot of folks who are more likely to have their own gardens, raise their own chickens or meat, or go hunting. So, there’s a lot more like self-reliance, I would say, on some more… nutritious foods.”

Public health/nutrition program staff member, Maine.

The participants noted that successful FSP requires adequate time, knowledge, finances, access to land, appropriate tools, and access to the licenses required to procure food (e.g., hunting and fishing licenses). They also discussed specific community assets that support FSP that currently exist and that they believe could be expanded or leveraged for additional impact, including community gardens, FSP educational programs, and grants that fund initiatives to increase access to FSP activities. Community gardens were reported to be common across the region and were described as an asset that allows more people to participate in FSP. Two participants noted that the public library in their area lends out fishing and gardening gear and distributes seeds so that residents can engage in these activities without having to purchase all the resources typically required.

Participants noted the importance of agriculture and farming in the region, which could have a positive effect on nutrition security. The benefit and value of programs and initiatives that increase access to local agricultural products was highlighted. Participants across all three states described gleaning efforts in rural areas designed to move surplus produce and foods from grocery stores and farms to food pantries. One participant noted that a seed company in their state will donate produce grown in its research fields that would otherwise go to waste.

“Here in Vermont… we’re very fortunate because we are still an agricultural state and granted there’s barriers but there is, during the growing season, access to a lot of fresh produce which is so incredibly important. And local, meat-based protein and dairy, a lot of dairy.”

Extension agent, Vermont.

“You know, there are spatterings of programs like that in rural areas in Maine, whether it’s… community, you know, programs to learn how to forage, learn how to garden… There’s gleaning programs… that get fresh food out of the fields, into food pantries or into school, you know, wherever it is.”

Food systems network staff member, Maine.

### 3.3. Social Capital/Human Capital

Social networks were identified as an asset for healthy food access due to their ability to both help community members in need and promote trust in nutrition interventions. Participants described how social networks can promote resilience against circumstances that might threaten nutrition security. Participants frequently mentioned rural residents’ tendencies to care for each other and support community members who are in need. In addition to discussing sharing food, participants mentioned other types of social support that can bolster nutrition security, such as transportation (e.g., delivering food to those without access to a vehicle), childcare, and peer support for physical activity. Two participants noted that a potential benefit of utilizing networks to acquire healthy food is the informality of these methods. Specifically, exchanging food through social networks bypasses traditional market structures, supporting the nutrition of individuals and households who may be unable to afford healthy food. Similarly, the lack of bureaucracy or paperwork needed to acquire food through social or informal networks was seen as a mechanism of supporting equity.

“I think that one of the beauties of Maine, too, is that… there really is a sense of community within individual communities and I think there tends to be a lot of taking care of each other. And what I love is, for example, in some communities, you drive down the road and there’s a little stand outside that says ‘free zucchinis’ or, you know, ‘free, help yourself’. I feel like, you know, that is one of the wonderful things about Maine and about… some of the rural aspects… of the state is that people tend to maybe, for example, grow a little more… to provide that support to their neighbors as needed.”

Food bank staff member, Maine.

Participants also discussed how social networks can help promote trust in nutrition programs, potentially improving program efficacy. Comments regarding social capital assumed that a certain level of social connection amongst community members had been established prior, providing a basis for actions that support nutrition security in the future. For example, one participant gave an anecdote about how participation in the Supplemental Nutrition Assistance Program’s (SNAP) Double Up Food Bucks program increased when community members were recruited to share information about the program with their social networks.

Collaboration and coordination across different aspects of the food system was identified as another asset related to human and social capital. This form of capital intersects with built capital, as it relates to both institutions and individuals. Participants noted that food pantries and food shelves operate at numerous non-traditional points of food access in rural communities, including churches, hospitals, schools, public libraries, family centers, help-yourself-shelves, youth organizations such as Boys and Girls Clubs, and older adult organizations such as day programs and senior housing centers. Participants mentioned how these organizations often coordinate and sometimes share surplus food or unneeded infrastructure. Similarly, participants discussed how small food businesses such as farm stores and local food distributors collaborate for mutual benefit, such as group buying to meet minimum purchasing requirements set by larger food distributors. This coordination is facilitated by the social networks and relationships between organizations.

“There’s a bunch of food pantries that communicate with each other. They, you know, they may share food… somebody may throw out like, ‘hey, I’ve got an extra refrigerator. Does anybody need it?”

Farm-to-school program staff member, New Hampshire.

### 3.4. Political Capital

Local political commitment to food and nutrition security is another potential asset. Participants described rural residents taking advantage of federal and state nutrition assistance programs including SNAP, the Special Supplemental Nutrition Program for Women, Infants, and Children (WIC), and SNAP’s Double Up Food Bucks program. Participants from Maine and Vermont emphatically shared the belief that the relatively recent implementation of universal school meals has had a positive impact on nutrition security, potentially due to its lack of a requirement for means testing.

“I’m so grateful that Vermont went to universal school meals for kids because so many families were just, like missing it by literally $5. And, you know, no matter what the school professionals were doing, they just couldn’t, you know… squeeze that peg into that round hole… So, you know, universal school meals is really helpful.”

Public health program staff member, Vermont.

Although New Hampshire does not have universal school meals, three participants from that state spoke of the perceived benefits for nutrition. For example, extension agents from Vermont and New Hampshire had the following exchange during one of the focus groups:

Extension agent from Vermont: “One of the strengths here of the community is that Vermont has universal free school meals, and that includes breakfast, lunch, and… some schools have a pretty robust like end-of-day snack, or it’s almost like a mini meal that kids can take home…”

Extension agent from New Hampshire: “I was just gonna say I wish we had that in our schools here. I just want that for all the kids.”

Both statewide and local Hunger Councils and food security-focused coalitions were highlighted for their role in facilitating coordination to support nutrition security and advocate for nutrition policy. Participants in New Hampshire and Vermont explained how these types of organizations help build political capital for nutrition.

“And the Food Access Coalition… much like Hunger Free Vermont, has a lot of partners at the table. They have monthly calls. There’s, you know, all kinds of people that attend that are involved in food and food access from state agencies to nonprofits and Farm to School and Extension, all participate… So, it’s… a network that shares information, we work on policy together…”

Farm-to-school program staff member, New Hampshire.

### 3.5. Financial Capital

While many participants emphasized a lack of financial resources in rural communities, a few noted that the regional tourist economy generates a significant amount of financial revenue for selected communities in NNE. Tourism supports the local economy and allows for a stronger rural economy in the region, potentially supporting economic access to food.

“During foliage season in the fall and wintertime, you’ve got more tourists coming through and they’re gonna spend money at the restaurants. They’re gonna tip, you know, tend to give bigger tips to the waiters and waitress staff. So, they’re going to have more money to spend on other things like food and all that.”

Health non-profit staff member, Vermont.

## 4. Discussion

In recent years, there has been increasing recognition of the fact that improving rural health and advancing equity will require leveraging strengths and resources that already exist in rural places [38,39]. Yet most rural health research continues to focus on rural risk factors and barriers to healthy behaviors. There also has been a push to explore rurality as a collection of heterogeneous communities with unique strengths and needs [40,41]. Stereotypical ideas of rural areas as “singular deficit-based places” as opposed to “nuanced, thriving spaces” [40] (p. 136) neglects the diversity of rural America and hampers innovation to promote nutrition. This novel study used an asset-based lens to identify factors that support nutrition security in rural NNE. By studying rural communities in three small, contiguous, and culturally similar states, this study sought to overcome some of the scale challenges of research that focuses on just one community or endeavors to represent all rural areas. By applying the CCF, we found multiple forms of community capital relevant to rural diets, especially natural, cultural, human, social, political, and, to a lesser degree, financial.

These results demonstrate the value of an asset-based approach and build on and expand the other research that has attempted to capture assets in rural communities relevant to health equity [39] and food and nutrition security specifically [31,42,43]. For example, in their work in rural Appalachia, Batey et al. [42] found that cultural factors such as the intergenerational sharing of cultural knowledge related to food; social and family networks; and strategies of resilience against reduced food access support food security. In a study in Michigan’s Western Upper Peninsula, Lu and Carter [43] found a web of interconnecting influences related to food security, with FSP and social networks being two positive contributing factors. Finally, Hardison-Moody et al. [31] found that the availability of grants, development of community coalitions, and presence of green spaces may be supportive of physical activity and healthy food access across four rural counties in North Carolina.

Food production has long been part of the cultural identity of NNE [44], with potential impacts on food choices. This was emphasized by participants in the present study. Compared to farms in other parts of the country, those in NNE are more likely to be fruit, vegetable, or dairy farms [45], indicating an opportunity for rural residents to access healthy foods that are local and meaningful to them. Local food sources, such as farmer’s markets and community-supported agriculture programs (CSAs), were found to be the second most utilized food source behind grocery stores amongst residents of Maine and Vermont prior to the COVID-19 pandemic [46]. Similarly, many participants noted the importance of FSP activities to the region’s food culture and as a source of nutrition. During the COVID-19 pandemic, 60% of households in Maine and Vermont reported participating in food self-provisioning activities, including gardening, food preservation, fishing, hunting, foraging, and raising livestock, and 80.7% stated they would engage in at least one of these activities the following year [47]. Despite the potential importance of FSP to rural diets in the region, it is not frequently considered in initiatives to improve nutrition security in rural areas [48], suggesting an opportunity for future work.

The results of this study regarding the power of rural social networks in the NNE align with prior studies that have emphasized the importance of social cohesion and relationships for supporting rural health [49]. For example, one study found that the exchange of produce through social networks can be useful in improving the diversity of the consumption of fruits and vegetables and reaching the recommended intake of vegetables [50], and another found that sharing meals was linked to improved food security [51]. Gathering over food is an integral aspect of social activities in rural areas, a feature that may serve to support or hinder healthy eating habits [52].

Participants in this study discussed high levels of collaboration and coordination between different aspects of the food system, with potential benefits for nutrition security. Previous studies have examined collaborative efforts between food pantries and food banks [53], as well as the importance of sharing information and resources for rural food policy councils [54]. Collaboration between different organizations may also help maximize the use of limited funds when working towards a common goal [31]. Additionally, human capital was found to be supportive of the development of the social capital of food bank supply chains [55]. Little research has specifically focused on the utilization of collaborative efforts in rural communities to improve nutrition security. Future research should consider this potential leverage point in the food system in greater depth. Social Network Analysis could be used to identify food and nutrition leaders in a community and key connections that do or could support nutrition security. For example, states’ Extension Services could use Social Network Analysis to identify those who could most effectively help spread messages about program offerings, ensuring that the information reaches the groups in greatest need and prompts participation.

Maine and Vermont are well known for their political commitment to food security [56,57], which was emphasized by experts in this study. In particular, participants discussed this topic in relation to universal school meals as well as civic engagement focused on food security, for instance, the work of Hunger Councils. Beyond what the participants mentioned, several other pieces of innovative food system legislation have been passed in recent years in NNE, including the constitutional Right to Food amendment [58] and the Food Sovereignty Act in Maine [59]; bans on sending organic waste to landfill in New Hampshire and Vermont [60]; and state government-funded incentive grants for local food purchasing in Vermont [61]. The high level of civic and political engagement related to food in the region, especially in Maine and Vermont, may be unique, but it also provides lessons for other rural regions regarding the potential impact of political capital for advancing nutrition security.

The discussion of financial capital was limited to a small number of participants who spoke about the seasonal tourist economy in selected communities in rural NNE. For these communities, tourism is a major source of income. According to the USDA Economic Research Service, over half of the non-metropolitan counties across the three states rely on recreation as their predominant source of economic output [62]. Tourism-linked cash flows may support healthy food access in some, but not all, seasons. Thus, communities may have opportunities to identify strategies that will maximize the benefits of this seasonal asset for year-round benefit.

The participants’ discussion of aspects of the built environment (referred to as ‘built capital’ in the CCF) that support healthy food access in rural NNE was limited to referencing different key institutions (e.g., food pantries). However, previous research has established a link between the built environment and rural nutrition security [63,64]. For example, the relationship between social and built capital was a crucial factor supporting community food security during the aftermath of a major flooding event in Vermont in 2011. Private infrastructure, such as local restaurants and grocery stores, and public infrastructure, such as schools, green spaces, and food pantries, offered communal spaces for the development of social capital and recovery efforts after the flood [63]. These results reflect those found in another study regarding the local food system and informal economy in the rural Midwest [64]. Mapping the connections between forms of capital in specific rural communities may help present a clearer picture of the assets they can employ to achieve nutrition security while highlighting the heterogeneity of rural communities and the value of place-based assessments.

The findings must be considered in light of the study’s limitations. First, reliance on purposive and snowball sampling allowed for identification of known food system experts, but these methods relied on the subjective judgements of the research team and may have contributed to limited representation of diverse perspectives. For example, most of the participants were white women, reflecting the demographics of nutrition and dietetics professionals nationally [65], but not necessarily the groups experiencing the greatest need. The participants in this study mentioned people of color, New Americans, Indigenous people, members of the LGBTQIA+ community, people living with disabilities, children and young families, older adults, and people who are unhoused and/or have a low income, among others, as groups who have an especially hard time accessing healthy food in rural communities, but participants did not necessarily represent these groups themselves. Indeed, marginalized communities have a disproportionately difficult time achieving nutrition security compared to members of nonmarginalized groups [2] and many face discrimination in their daily lives and additionally when accessing healthy foods. These groups also have their own assets and strategies for resilience that they employ to acquire healthy foods that are meaningful and relevant to them, which may not have been captured by this study. More research including these communities is needed to understand their unique resources for nutrition security.

## 5. Conclusions

This study found a variety of assets that support nutrition security in rural NNE that may be overlooked in traditional assessments of healthy food access. The uncovering of unique assets—for example, those related to political capital—reinforces calls for place-based assessments. The findings are specific to NNE and cannot be generalized to other rural populations where preferred foods, customs, and social structures may differ. The study sample comprised food system experts, but did not reflect the rural groups in greatest need of nutrition support. Future research and programming on rural diet quality in NNE should account for and build upon the identified assets and community strengths, including discussing and reflecting on the findings of research involving vulnerable rural populations. Asset-based approaches may benefit from integration with research tools designed to examine relationships, such as Social Network Analysis. Leveraging the identified resources may provide an opportunity to advance rural health equity through more nutritious diets.

## Figures and Tables

**Table 1:** Participant characteristics, 2023–2024.

Characteristic	Participants (*n* = 32)
Age	
Range	24–66
Median	45
Employer or profession (%)	
Public health/nutrition program	34.38
Food or farm non-profit organization	28.13
University Extension agent	18.75
Food hub	9.38
Clinical dietitian	6.25
Indigenous community leader	3.13
Gender (%)	
Female	93.75
Male	6.25
Race (%)	
Non-Hispanic White	91.63
Black	6.25
Native American	3.13
State (*n*)	
Maine	10
New Hampshire	7
Vermont	15

## Data Availability

To maintain the privacy of the participants, the data presented in this study are available on request from the corresponding author.

## Supplementary Materials

The following supporting information can be downloaded at: https://www.mdpi.com/article/10.3390/nu17020295/s1, Supplementary S1: Focus Group Discussion Guide, Supplementary S2: Interview Discussion Guide, Supplementary S3: Code List. Reference [33] is cited in both Supplementary S1 and S2.

## Abbreviations

The following abbreviations are used in this manuscript:

USDA	U.S. Department of Agriculture
NNE	Northern New England
CCF	Community Capitals Framework
FSP	Food self-provisioning
SNAP	Supplemental Nutrition Assistance Program
WIC	Special Supplemental Nutrition Program for Women, Infants, and Children
CSA	Community-supported agriculture program
