# Supplementary material for: An Asset-Based Examination of Contextual Factors Influencing Nutrition Security: The Case of Rural Northern New England
Source: Nutrients. 2025 Jan 15;17(2):295. doi: 10.3390/nu17020295 (PMC11767827; doi:10.3390/nu17020295)
Supplement: Supplementary file 1 [file nutrients-17-00295-s001.zip › nutrients-3417657-supplementary.pdf]

Supplementary Files

S1. Discussion Guide for Focus Groups

Introduction

Thank you for joining me for this focus group discussion.

My name is [NAME], and I am a research assistant working with Dr. [NAME] at the University of Vermont. As you've heard in our other communications, the goal of this focus group is to generate dialogue on the dietary patterns of rural northern New Englanders. In some ways, this is like a collective brainstorming session. There are no right or wrong answers, and it is okay to disagree with people, as long as you do so respectfully. In fact, we hope to hear lots of different opinions and perspectives. To maintain this mutual respect, we ask that you not repeat what you hear others say outside of this discussion. After this discussion has concluded, our next step will be deidentifying the discussion transcript, so your identity will be anonymous for all future steps of the research process.

Through this focus group and others like it, we specifically want to identify some of the characteristics that distinguish rural communities from each other and delve into the qualities that may contribute to the consumption of a nutritious diet. In particular, we are looking to identify social, financial, physical, environmental, or interpersonal resources in rural communities that support the adoption and maintenance of high-quality diets. We would like to use an asset-based approach to our discussion today. This means we will seek to identify the factors that promote diet quality, including a community's strengths and strategies for resilience, instead of just focusing on the barriers or factors that negatively influence it. Previous research by our team at UVM has found that – like many Americans in the rest of the U.S.– most people in our region eat far too few recommended foods like vegetables, fruits, and whole grains – and high levels of things like added sugars that the national guidelines suggest limiting. Using the results from our discussion today, we hope to understand what community-level factors promote a healthy diet in rural Northern New England regions.

As I mentioned previously, we will be recording today's session so that a transcript of our discussion can be created for analysis. Before we continue to our discussion and I press record, I want to check if there are any questions?

Alright. Let's dive in. To begin, I'd like for everyone to introduce themselves and give us a very brief description of your job.

Thank you. Throughout this discussion, I want you think about the rural communities where you work. If you also happen to live in a rural community, you may take into account that community when responding to our questions, as well. If you talk about a community that is different from the one you work in, please identify it in your response.

Discussion

Community and Environmental Factors

- In the community where you work, what are the major fields or sectors that people are employed in?
  - Would people in the community where you work say that the local economy is healthy or struggling?
- From a nutrition perspective, what populations would you describe as more vulnerable or marginalized in the communities where you work?
  - As we continue this discussion, I request that you include these groups, and their specific circumstances in your answers to my upcoming questions.
- Are there any local traditions or customs around growing food, acquiring food, cooking, or eating that stick out to you as supporting a nutritious diet? What are the main ways that people access food in the communities where you work?
  - PROBES: grocery stores, farmers' markets, free or emergency food programs, gardens, hunting, etc.
  - How do people with more nutritious diets access food?
    - PROBES: geography or proximity to stores/restaurants/food pantries
  - How do people with less nutritious diets access food?
- Is there anything unique about the food scene in the community where you work that is different from what you see in mainstream media?
- What are some characteristics of the community or physical environment in your area that you think support a healthy diet?
- What are some of the local health/nutrition organizations and what are the community's perceptions of them?
  - PROBES: non-profit groups, government programs, social enterprises
- Are there other community groups that you feel support a nutritious diet?
- Are there any community-wide activities that take place in your area that you feel support a nutritious diet?

Household Characteristics and Behaviors

- What networks exist that might promote the consumption of a healthy diet?
- Can you think of any traits of people who consume a healthy diet despite facing the same barriers as their fellow community members? Please describe some of these qualities.

Rural Categories

- Next, I am going to define three categories of rurality that have been pulled from a framework created by social science researchers Dr. Cynthia Duncan at the University of New Hampshire and Dr. Jessica Ulrich-Schad at Utah State University. The three categories are amenity-rich areas, transitioning areas, and chronically-poor areas. As I am reading these definitions, I would like for you to consider how the communities where you live and/or work fall within these categories. Please keep in mind that these definitions may not

perfectly describe your communities and that's okay - there can be nuance in these categorizations.

- The first category is amenity-rich areas, which are popular for tourism and recreation due to their natural amenities. These areas have seen an increase in population growth as more retirees and college-educated professionals move into these areas.
- The second category is transitioning areas, which have regional economies based around agriculture, timber, and/or manufacturing. Their population trends are in flux, with some communities experiencing increases in their population while others are experiencing decreases.
- The third and final category is chronically-poor areas, which have experienced poor economic prospects for an extended period of time. These regions have seen a long-term lack of investment in education and other community services while having experienced significant population decline over the years.
- What are some of the communities in your state where you live and/or work and how would you categorize them using these definitions?
- How do you feel your community's categorization affects its members' abilities to consume a nutritious diet?
    - PROBES: shifts in the dominant economic sectors or socioeconomic status of the region, changes in the reliability in social institutions, changes in the senses of pride or cohesion in the community, changes over time, etc.

Subgroups

- For our final discussion topic, I would like to ask you to reflect on the vulnerable populations that we talked about before. Are there any "groups of people" that you feel have an especially hard time consuming a healthy diet? Can you tell me about those groups?
    - What are some of the circumstances they face that make this so difficult?
- Now thinking about the opposite, are there any "groups of people" that you feel are successful in achieving a healthy diet? Can you tell me about them?
    - PROBES: attributes that contribute to success, strategies that contribute to success, environmental factors that contribute to success

Conclusion

Before we finish, does anyone have any final thoughts or remarks that they would like to add? This is a great time to let us know if there is anything relevant that didn't come up during the questioning, but that we should know about the people that you work with and the foods they eat.

If you think of anything else you'd like to add that wasn't mentioned today, please feel free to contact me at [email address].

Thank you again for your time and input.

S2. Discussion Guide for Interviews

Introduction

Thank you for joining me for this interview.

My name is [NAME], and I am a research assistant working with Dr. [NAME] at the University of Vermont. As you've heard in our other communications, the goal of this interview is to learn more about contextual factors that contribute to the dietary patterns of rural northern New Englanders.

With this interview and others like it, we specifically want to identify some of the characteristics that distinguish rural communities from each other and delve into the qualities that may contribute to the consumption of a nutritious diet. In particular, we are looking to identify social, financial, physical, environmental, or interpersonal resources in rural communities that support the adoption and maintenance of high-quality diets. For clarification, when I use the word communities, I am referring to both geographic and social communities. We would like to use an asset-based approach to our discussion today. This means we will seek to identify the factors that promote diet quality, including a community's strengths and strategies for resilience, instead of just focusing on the barriers or factors that negatively influence it.

As I mentioned previously, we will be recording today's session so that a transcript of our discussion can be created for analysis. When we finish, I will remove your name from the transcript to help protect your confidentiality during the analysis and reporting processes. Before we continue to our discussion and I press record, I want to check if there are any questions?

Alright. Let's dive in. To begin, would you give me a brief description of your job and/or your connection to the regional food system.

Discussion

Community and Environmental Factors

- From a nutrition perspective, what populations would you describe as more vulnerable or marginalized in rural communities in your region?
    - What are the circumstances these groups face that make consuming healthy food more difficult?
- In what ways do you think living in a rural area shapes what people eat, for better or worse? How do certain factors (environmental, cultural, social, economic, etc.) affect rural residents' abilities to consume a healthy diet?
    - PROBES
        - ENVIRONMENT: built environment, natural environment
        - CULTURE: local traditions or customs, specific perceptions of or attitudes towards food, common habits amongst residents related to food or diet
        - SOCIAL/COMMUNITY: networks, other social or community-related activities or qualities
        - ECONOMY: rural economic health, major economic sectors

    - FOLLOW UP: how are different communities in your state affected by these factors? Do these factors affect certain types of communities differently?

Rural Categories

- Reviewing your responses to the rural categories worksheet, can you talk me through your reasoning behind these categorizations?
- I would like to talk about how a county's categorization might affect to its residents' abilities to consume a healthy diet. Do you have any thoughts on the relationship between these categorizations and diet?
    - PROBES: shifts in the dominant economic sectors or socioeconomic status of the region, changes in the reliability in social institutions, changes in the senses of pride or cohesion in the community, changes over time, changes in population, etc.
- Given what we've just discussed, how would you rank economic factors against other factors in terms of affecting the healthfulness of rural diets?
    - Can you tell me more about that?

Conclusion

[Follow up on any points that weren't fully explained or could use clarification].

Before we finish, do you have any final thoughts or remarks you would like to add? This is a great time to let us know if there is anything relevant that didn't come up during the questioning, but that we should know about rural diets in your state.

If you think of anything else you'd like to add that you didn't mention today, please feel free to contact me at [email address].

Thank you again for your time and input.

S3. Analytical Codes and Most Salient Links to the Community Capitals Framework

CODE	DEFINITION	CCF CONNECTIONS
A. Built or natural environment		
A1. Proximity to food stores	Comments relating to proximity to stores where residents purchase food	Built capital<br>Natural capital<br>Financial capital<br>Political capital
A2. Land access	Comments relating to access to land for living, farming, or other forms of food acquisition	Natural capital<br>Financial capital<br>Cultural capital<br>Political capital
A3. Types of food stores	Comments relating to the types of food outlets/stores that residents rely on	Built capital<br>Financial capital<br>Political capital
B. Economy		
B1. Cost of living	Comments relating to budgeting/affording necessities (i.e. utilities, housing, healthcare)	Financial capital<br>Political capital
B2. Cost of food	Comments relating to food prices or the ability to afford food	Financial capital<br>Political capital
B3. Socioeconomic status/job availability	Comments relating to socioeconomic status or the availability of jobs in a region	Financial capital<br>Political capital<br>Human capital
C. Culture		
C1. Food or health-related attitudes/perceptions	Comments relating to values or perceptions regarding health, food, food systems	Human capital<br>Cultural capital
C2. Home food production/acquisition	Comments relating to activities in which people acquire or produce food at home (i.e. gardening, canning, hunting)	Cultural capital<br>Natural capital<br>Human capital
C3. Culturally meaningful foods	Comments related to any food items, food-related practices, or cuisines	Cultural capital<br>Natural capital<br>Built capital
D. Institutions		
D1. Barriers to accessing food or nutrition services	Comments relating to structural/institutional barriers to healthy food access	Human capital<br>Political capital<br>Financial capital
D3. Transportation	Comments relating to residents' access or use of any form of transportation	Built capital<br>Financial capital<br>Political capital
E. Marginalized communities		
E1. Marginalized communities	Comments including mention of any marginalized community	N/A

E2. Circumstances that marginalized communities face	Comments including mention of circumstances faced by any marginalized community that impacts access to healthy food	Human capital<br>Political capital<br>Financial capital
F. Social factors		
F1. Community gatherings	Comments referring to community-wide events	Social capital<br>Cultural capital<br>Built capital
F2. Social support networks	Comments relating to friends, neighbors, family providing food or other forms of support for each other	Social capital<br>Human capital
F3. Stigma	Comments relating to perceptions of stigma related to food, public assistance programs, etc.	Cultural capital<br>Financial capital
G. Organizations		
G1. Government programs or policies	Comments mentioning programs established or funded by the government that provide access to free or reduced-price food	Political capital<br>Financial capital<br>Human capital
G2. Non-profits	Comments mentioning non-profit organizations that support access to healthy foods	Built capital<br>Human capital
G3. Social enterprises	Comments mentioning programs or policies focused on improving the access or affordability of healthy foods that are established/run by for-profit companies	Financial capital<br>Built capital
H. Personal factors		
H1. Education, knowledge, skills, time, motivation	Comments related to having the education, knowledge, time, motivation to eat healthily	Human capital
I. Rurality framework		
I1. Amenity-rich areas	Comments identifying a region as having tourism and recreation due to their natural amenities. These areas have seen an increase in population growth as more retirees and college-educated professionals move into these areas	Natural capital<br>Financial capital<br>Human capital<br>Political capital
I2. Transitioning areas	Comments identifying a region as having regional economies based around agriculture, timber, and/or manufacturing. Their population trends are in flux, with some communities experiencing increases in their population while others are experiencing decreases	Financial capital<br>Political capital

I3. Chronically poor areas	Comments identifying a region as having poor economic prospects for an extended period of time. These regions have seen a long-term lack of investment in education and other community services while having experienced significant population decline over the years	Financial capital<br>Political capital<br>Human capital
